# The Direct 3D Printing of Functional PEEK/Hydroxyapatite Composites via a Fused Filament Fabrication Approach

**DOI:** 10.3390/polym13040545

**Published:** 2021-02-12

**Authors:** Krzysztof Rodzeń, Preetam K. Sharma, Alistair McIlhagger, Mozaffar Mokhtari, Foram Dave, David Tormey, Richard Sherlock, Brian J. Meenan, Adrian Boyd

**Affiliations:** 1 School of Engineering, Ulster University, Shore Road, Newtownabbey, Co. Antrim BT37 0QB, Northern Ireland, UK; pk.sharma@ulster.ac.uk (P.K.S.); a.mcilhagger@ulster.ac.uk (A.M.); m.mokhtari@ulster.ac.uk (M.M.); bj.meenan@ulster.ac.uk (B.J.M.); 2 Centre for Precision Engineering, Materials and Manufacturing Research, Institute of Technology Sligo, Ash Lane, F91 YW50 Sligo, Ireland; Foram.Dave@mail.itsligo.ie (F.D.); Tormey.David@itsligo.ie (D.T.); Sherlock.Richard@itsligo.ie (R.S.)

**Keywords:** additive manufacturing, advanced composite materials, 3D printing, fused filament fabrication, PEEK, polyetheretherketone, hydroxyapatite

## Abstract

The manufacture of polyetheretherketone/hydroxyapatite (PEEK/HA) composites is seen as a viable approach to help enhance direct bone apposition in orthopaedic implants. A range of methods have been used to produce composites, including Selective Laser Sintering and injection moulding. Such techniques have drawbacks and lack flexibility to manufacture complex, custom-designed implants. 3D printing gets around many of the restraints and provides new opportunities for innovative solutions that are structurally suited to meet the needs of the patient. This work reports the direct 3D printing of extruded PEEK/HA composite filaments via a Fused Filament Fabrication (FFF) approach. In this work samples are 3D printed by a custom modified commercial printer Ultimaker 2+ (UM2+). SEM-EDX and µCT analyses show that HA particles are evenly distributed throughout the bulk and across the surface of the native 3D printed samples, with XRD highlighting up to 50% crystallinity and crystalline domains clearly observed in SEM and HR-TEM analyses. This highlights the favourable temperature conditions during 3D printing. The yield stress and ultimate tensile strength obtained for all the samples are comparable to human femoral cortical bone. The results show how FFF 3D printing of PEEK/HA composites up to 30 wt% HA can be achieved.

## 1. Introduction

Polyetheretherketone (PEEK) is a high-performance thermoplastic polymer of particular interest for replacing metals in orthopaedic implant applications due to its excellent biocompatibility, radiolucency, low specific gravity (1.3 g/cm^3^), and its favourable mechanical properties [1]. Further to this it has a lot of potential for ‘made-to-measure’ implants produced through additive manufacturing (AM) approaches, namely 3D Printing (3DP) [2,3]. Such an approach enables the development of precision biomaterials and is a critical enabling technology for their clinical implementation [4]. However, PEEK is hydrophobic due to the aromatic rings and polyester functional groups backbone structure, which typically inhibits cell attachment and leads to poor bone apposition [5]. Consequently, there is a need to provide a mechanism to functionalise its surface to make the material at least osteoconductive to ensure a more rapid, improved, and stable fixation that will last longer, in vivo. One approach to solving this issue is to modify PEEK with bioactive calcium phosphate (CaP) materials, such as hydroxyapatite (HA). This could be achieved via coating the PEEK with HA [6,7] or manufacturing a PEEK/HA composite material and ensuring that the bioactive HA component is available on the surface of the material to deliver the desired osseointegration [8].

There is a significant body of work in the literature that describes how PEEK and HA can be combined to create composite structures for orthopaedic implant devices [9]. Recent work by Ma et al. reports how compounding and injection moulding can be used to create PEEK/HA composite materials, highlighting the favorable in vitro properties achieved by up to 40% loading by weight of HA in PEEK, with optimal results reported at 30% weight loading of HA [10]. Other approaches have also been used, including extrusion free forming in combination with compression moulding [11,12], selective laser sintering [13], cold press sintering [14], hot pressing to create a functionally graded material [15] and electrostatic bonding [16]. In other approaches PEEK implant structures were created by subtractive manufacturing approaches, which generated a lot of waste material and is known to have limited scope for preparing complex geometries when compared to other techniques such as 3D printing [17,18]. Notably, 3D printing gets around many of the restraints in traditional manufacturing techniques and opens up opportunities to provide novel and innovative solutions that are structurally suited to meet the needs of the patient. When it comes to 3D printing of PEEK, techniques such as Selective Laser Sintering (SLS) and Fused Filament Fabrication (FFF) have been successfully utilised and can provide solutions to some of the issues with traditional manufacturing approaches highlighted above. SLS has been successfully applied to build a 3D printed PEEK replacement for a scapula containing an invasive primary bone tumour [19]. The optimization of the SLS printing process was also investigated on cranial implants, to help reduce any anisotropy within the printed body [20]. However, the SLS technique is very high cost and also results in significant waste of the PEEK powder, which cannot be re-used for implant preparation thereafter due to the potential for powder contamination. In the SLS approach, the PEEK powder is also difficult to handle, requiring additional safety measures. The second technique, FFF utilises a continuous filament of PEEK during 3D printing. The process produces minimal waste, is an easy and accessible technology, and can undergo significant modification to develop printers better suited for an intended application area. One major drawback of FFF for 3D printing of PEEK is the subsequent anisotropy of the mechanical properties in prints, and the requirement for the chamber, print bed and hot-end temperatures to be high, typically well beyond those available in normal commercial FFF printers [21,22,23,24]. As such, the properties and subsequent performance of semi-crystalline PEEK materials produced through FFF 3D printing will strongly depend upon providing the correct processing parameters during printing. It is hypothesized that these could be carefully modified and controlled to minimise the normal inconsistency observed in their mechanical properties by allowing fine-tuning of the crystalline microstructure and would ultimately provide a direct and cost-effective route for 3D printing of PEEK/HA composites [25].

In this paper we would like to present a significant development in 3D printing of PEEK and HA composites using an FFF approach. The key aim of the work was to prove that FFF 3D printing could deliver PEEK/HA composites with mechanical properties matching those of bone, and, to the best of our knowledge, this is the first time this has been reported in the literature. Such an approach could be used to easily manufacture suitable print geometries via a Point of Care Medical Manufacturing (POCMM) approach to develop made-to-measure implants for applications such as spinal fusion, without all the associated drawbacks of FFF [26]. In this work we report firstly on the creation of PEEK/HA composite filaments via twin-screw extrusion, and the subsequent direct 3D printing of the extruded PEEK/HA composite filaments via a FFF approach using a custom modified commercial printer Ultimaker 2+ (UM2+). The 3D printer was modified to operate with the print chamber temperature up to 230 °C, the print bed up to 320 °C and the hot-end printing nozzle up to 420 °C, allowing the crystal structure of the printed PEEK bodies to be modified accordingly, which has a subsequent influence on the properties of the resultant printed bodies. The 3D printed specimens were then subject to characterisation via mechanical, thermal, physical, and chemical techniques (as detailed in the materials and methods section) to ascertain the potential for FFF to be a go-to manufacturing technique to produce PEEK/HA composites for orthopaedic implant devices, where direct bone apposition is crucial.

## 2. Materials and Methods

### 2.1. Materials and Processing Conditions

The CAPITAL^®^R (Plasma Biotal, Buxton, UK) unsintered Hydroxyapatite powder (HA) was mixed with medium viscous polyetheretherketone (PEEK) VESTAKEEP^®^ 2000P (Evonik, Essen, Germany) and processed by twin-screw extrusion to obtain continuous filaments with five different HA contents: 0, 5, 10, 20, 30 wt% PEEK/HA and a continuous diameter of 1.75 ± 0.10 mm, enabling the filament to be rolled onto a reel for 3D printing. PEEK and HA powders were dried in an oven at 170 °C for 12 h before use and premixed at various compositions. A co-rotating twin-screw extruder (Rheomex PTW16/40 OS) with L/D = 40 and diameter of 16 mm was utilised to compound the PEEK and HA into filaments with a diameter of 1.75 mm. It was operated at a screw speed of 45 rpm and an exact melt temperature of 370 °C which was measured in the die. An in-house screw configuration was used to distribute and disperse the HA powder in PEEK. The screw elements were constituted of feed screw elements for the forward and reverse conveying of materials in the feeding, conveying, reverse, venting and extrusion parts of the extruder and mixing elements of 90° and 0° for providing 30°, 60° and 90° twist angles for the melting and mixing parts of the extruder. (These materials were used for 3D printing on a modified UM2+ supplied with an all-metal hot end capable of reaching temperatures of up to 420 °C, building the with heating bed allowing regulation of the temperature up to 350 °C, and heating lamps for regulation of chamber temperature up to 230 °C as had been described in previous work [27]. All specimens were printed as solid, fully filled structures. Printing conditions were optimised with each sample to avoid warping of the sample, with the printing described in Table 1.

### 2.2. Characterisation of the Extruded Filaments and Composite Samples

The extruded filaments were only characterised using Thermal Gravimetric Analysis (TGA) and micro-Computed Tomography (µCT) to ascertain the distribution and content of HA in the filaments prior to 3D printing. The 3D printed samples were characterised using X-Ray Diffraction (XRD), Transmission Electron Microscopy (TEM), Scanning Electron Microscopy (SEM), Energy Dispersive X-Ray Analysis (EDX), Differential Scanning Calorimetry (DSC), Dynamic Thermomechanical Analysis (DMTA), micro-Computed Tomography (µCT), and tensile and flexural mechanical analyses. All details of these analyses are provided below.

*X-Ray diffraction (XRD)* measurements were performed with a PANalytical X’Pert PRO system, (Malvern Panalytical, Malvern, UK) using Cu K_α_ radiation (λ = 1.5406 Å) in 2θ range of 15–40°. The grain size was calculated from the Scherrer equation Equation (A1), Appendix A. The crystallinity was determined as the area of crystalline peaks divided by the sum of the areas of crystalline and amorphous peaks multiply by 100.

*Transmission electron microscopy (TEM)* and *selected area electron diffraction pattern (SAED)* measurements were performed using a field emission JEOL 2100F, (JEOL Ltd., Tokyo, Japan) microscope operated at 200 kV. The samples for TEM analysis were sliced with thicknesses of 30 µm using a microtome fitted with a diamond knife and lifted on to carbon coated copper grids. The samples were then dried in the oven overnight, with air circulation, at 60 °C.

*Scanning electron microscopy (SEM)* with *energy dispersive X-ray (EDX)* analysis of the 3DP samples was performed using a Hitachi SU5000 field emission instrument equipped with an X-Max^N^ 80 mm^2^ silicon drift detector (Oxford Instruments, Abingdon, UK). Images and EDX spectra were obtained at an accelerating voltage of 10 KV. To make the surface of the samples conductive for the electron microscopy, they were coated with a thin layer (~20 nm) of Au/Pd (60/40 ratio) using an Emitech K500X sputtering deposition with Ar gas. The SEM images of illustrating the print line diameters were measured using the same SEM system in Backscattered Electron mode (BSE), whereby the samples were not coated with a conductive layer. The accelerating voltage used was 20 KV and images were collected at a pressure of 50 Pa. 

*Dynamic Thermomechanical Analysis (DMTA)* were performed with a Q800 DMTA (TA Instruments, NJ, USA) with clamp dual cantilever in ramp temperature mode with 1 Hz and an oscillation amplitude 15 µm, using a heating rate of 10 °C/min in the rage of 50–330 °C, to yield the storage moduli (E″), loss moduli (E′) and the Tan δ (equal to the ratio E″/E′ and relating the energy dissipation relative to the energy stored in the material). The glass transition temperature (T_g_) was taken to be the temperature at the peak of the Tan δ curve. The DMTA rectangular specimens were 56 mm in length, 10 mm in width and 3.2 mm thick. Three specimens from each content were tested after printing.

*Thermal Gravimetric Analysis (TGA)* Mass-loss/temperature curves were obtained with a SDT Q600D thermogravimetric analyser (TA Instruments, NJ, USA). The 10 mg samples were measured at a heating rate of 10 °C/min under air as the purge gas in the range of temperature 50–800 °C.

*Tensile properties* and *flexural properties* were determined using the Instron 5500R Model (Instron Limited, Norwood, UK) at room temperature. The 3DP specimens were evaluated from specimens designed according to the ASTM 638 Type IV and ISO 178 Flexural standards. 5 specimens were manufactured and tested using both tensile and flexural experiments for each PEEK/HA wt% category.

*Differential Scanning Calorimetry (DSC)* (type, company, city, country) analyses were performed using a DSC Q100 (TA Instruments, NJ, USA). The instrument was calibrated using pure indium. The 5 mg sample was cut from the middle of rectangular specimen with 56 mm in length, 10 mm width and 3.2 mm thickness. The ramp temperature mode was used with a heating rate of 10 °C/min in the rage of 50–400 °C. The absolute crystallinity of the samples is calculated in accordance with Equation (A2), Appendix A.

*Micro Computed Tomography (µCT)* (type, company, city, country) scans were measured on a Microtomograph SkyScan 1275 (Bruker, Billerica, MA, USA), with Source Voltage 40 kV and Source Current 250 µA. The Image Pixel Sizes were equal to 5 µm and 8 µm for the extruded filaments (ϕ 1.75 mm) and rectangular (56 × 10 × 3.2 mm) specimens, respectively. The larger pixel size was due to the greater distance between the X-ray source and the specimen when a larger sample was measured. 

*Statistical Analysis:* The Tensile Modulus, Ultimate Tensile Strength, Tensile Yield Strain, Flexural Modulus, Ultimate Flexural Modulus and Line Diameter are reported as the mean ± standard deviation (where N = 5).

## 3. Results

### 3.1. PEEK/HA Distribution

The extruded filaments were characterised using Thermal Gravimetric Analysis (TGA) and micro-Computed Tomography (µCT) to ascertain only the distribution and content of HA in the filaments prior to 3D printing. The physical, chemical, thermal, and mechanical properties of the 3D printed samples were characterised using X-Ray Diffraction (XRD), Transmission Electron Microscopy (TEM), Scanning Electron Microscopy (SEM), Energy Dispersive X-Ray Analysis (EDX), Differential Scanning Calorimetry (DSC), Dynamic Thermomechanical Analysis (DMTA), micro-Computed Tomography (µCT), and tensile and flexural mechanical analyses.

**Figure 1:**
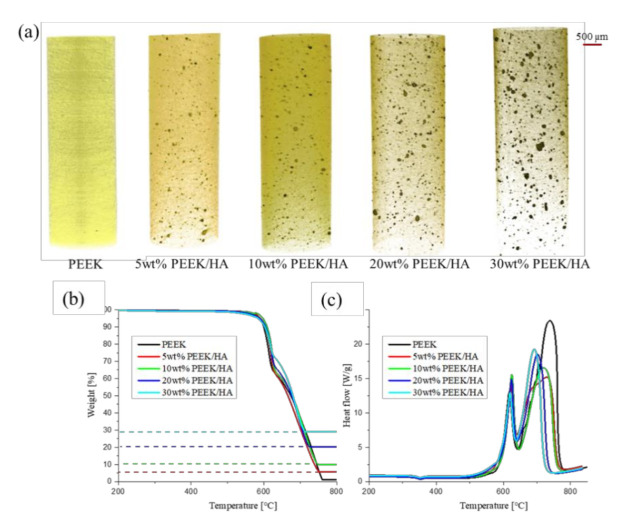
(**a**) CT Scans of produced filaments, (**b**) Thermogravimetry for the PEEK/HA composite filaments and pure PEEK with a heating rate 20 °C/min; weight% versus temperature and (**c**) Thermogravimetry heat flow versus temperature for the PEEK/HA composite filaments and pure PEEK.

**Figure A1:**
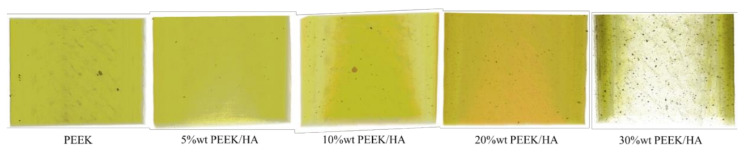
The CT Scans of 3DP rectangular specimens (pixel size 8 µm).

Micro Computed Tomography (µCT) scans show the good distribution of the HA inside the extruded filaments, as well in the 3DP specimens, as highlighted in Figure 1a and Figure A1 (Appendix A) for weight loadings of between 5 and 30% HA in the PEEK. The main fraction of the particle diameters observed are between 28 and 72 µm in both the filaments and the 3D printed samples. The pixel size is equivalent in this case to 5 µm, therefore smaller objects cannot be observed here by this technique. Larger agglomerates of HA particles can be observed in the µCT scans, and these can measure up to 179 µm in diameter. No HA particles or agglomerates were visible in the pure PEEK 3D printed samples (or the pure PEEK filament). It can be noted from the µCT results for the PEEK/HA filaments shown in Figure 1a that the 30 wt% PEEK/HA shows significantly larger HA agglomerates than the other samples. There are no visible changes in the size or distribution of the HA particles and agglomerates after the composite filaments are 3D printed, with both sets of samples showing identical properties via µCT analysis. The filaments used for the 3DP process were produced as solid and continuous filaments, with minimal porosity as reported in Table A1 (Appendix A). 3D printing of this continuous filament brought about a slight increase in the sample porosity, but this was observed to be well below 1% of the total volume, as highlighted in Table A1 (Appendix A) and as is a consequence of the FFF 3D printing technique, whereby the material to be printed is laid down in lines throughout the test specimen.

### 3.2. Thermal and Mechanical Properties

Thermogravimetric analysis (TGA) of the PEEK/HA composite filaments confirms the expected composition (0–30% HA by weight) was maintained during extrusion, as shown in Figure 1b. Leftover HA, remaining after oxidative decomposition of the organic fraction corresponds very closely to the amount of the HA mixed with PEEK in the preparation of the samples. The TGA heat flow curves, as shown in Figure 1c, highlight an endothermic melting peak at 343 °C for both the PEEK and PEEK/HA composites as would be expected. In the same figure, the oxidative decomposition of the PEEK and composite material can be seen to start above 500 °C and is largely unaffected by the HA content of the composite. However, the slope of the heat flow curves is greater as the weight content of HA increases (from 0–30% by weight). The first exothermic peak observed (between 500 °C and 650 °C) corresponds to the chain scission of the ether and ketone bonds, and oxidation, which is followed by the carbonization process. Carbonization by-products are further oxidized and visible as a second exothermic peak above 700 °C [28,29]. When the HA content is lower in the composite material the exothermic peak is shown to be broader and shifted to slightly higher temperatures. From these TGA results it appears that increased HA content in the composite filaments slightly reduces the activation energy of this process, but ultimately does not influence PEEK carbonisation. The reduction in the activation energy has also been previously observed for other polymer matrix hydroxyapatite composites [30].

**Figure 2:**
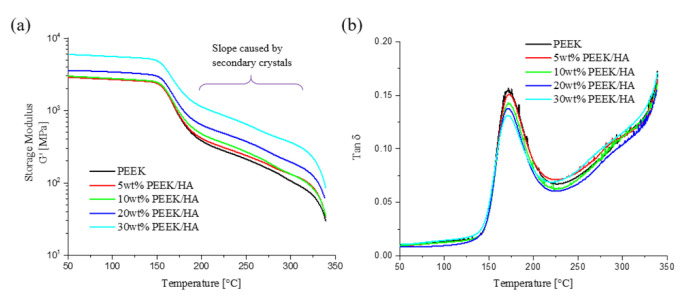
DMTA profiles for 3DP specimens: (**a**) Storage modulus vs. temperature, (**b**) *Tan δ* vs. temperature.

The Dynamic Mechanical Thermal Analysis (DMTA) profile for the 3D printed samples highlight that the storage modulus in the glass and rubber regions rise with increasing HA content, as shown in Figure 2a. The storage modulus at 100 °C of the PEEK sample is equal to 2736 MPa, whereas this increases to 5566 MPa for the 30 wt% HA loaded PEEK sample. From these results it is possible to distinguish a few reinforcement mechanisms for the PEEK/HA 3DP composites, especially for the 30 wt% HA loaded PEEK material. Firstly, incorporation of the HA filler into the PEEK leads to a reduction of the chain mobility and more reinforcement, thus increasing the storage modulus. As the fraction of HA increases (from 0–30% by weight) the scale of this phenomenon increases. This behaviour was largely expected, as the HA materials used in the composite formulation is characterized by an elastic modulus in the range of 80–120 GPa [31]. This is clearly observed as a gradual reduction of the *Tan* δ peak around 170 °C, which corresponds to a glass transition where the polymer chains can relax, as highlighted in Figure 2b [32,33].

The 3D printing process allowed deposited single lines of the material to be individually annealed, thus the layers created by those lines in the preheated chamber up to 230 °C allowed the formation of a well-defined crystalline (or semi-crystalline) printed structures. It is known that PEEK has a double melting behaviour, which is the result of melting of weak secondary and strong primary crystallites [34]. The primary crystallites are characterised by a higher melting temperature observed around 343 °C. The secondary crystallites can emerge between the glass transition temperature and melting point as an additional fraction that can be generated by annealing. The half-time crystallization study showed that the lowest value is reached here at around 230 °C, which corresponds to the temperature used in the 3D printing chamber [35].

**Figure 3:**
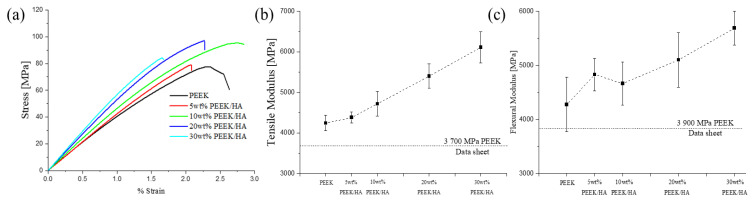
Mechanical properties of the samples: (**a**) tensile strength, (**b**) tensile modulus, and (**c**) flexural modulus.

**Figure A2:**
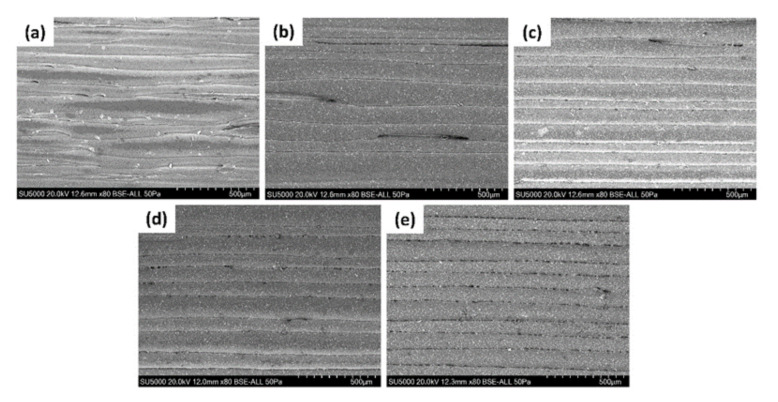
Cross-sectional BSE SEM images of 3D printed (**a**) pure PEEK, (**b**) 5 wt% PEEK/HA, (**c**) 10 wt% PEEK/HA, (**d**) 20 wt% PEEK/HA and (**e**) 30 wt% PEEK/HA.

Tensile tests of the 3D printed specimens showed their brittle character where the strain does not exceed 3%, as highlighted in Figure 3a and Table 2, with a maximum strain at break recoded at 2.7% for the 10 wt% HA loaded PEEK composite. This value for strain is low and is likely a consequence of the degree of crystallinity found in these 3D printed materials. This high degree of crystallinity would be expected due to printing conditions utilised in the 3D printing chamber, which should promote crystallization in these samples. It should also be noted that the 3D printed samples have a higher degree of porosity than the extruded filaments that were used to undertake the 3D printing, as seen in Table A1 (Appendix A), which was calculated from the µCT data. This would also affect the mechanical properties of the 3D printed samples. The porosity observed in all 3D printed specimens is a consequence of pores formed between lines/layers due to the imperfect deposition process that arises from FFF, as shown in the SEM images of cross sections of the samples in Figure A2 (Appendix A). The 3D printed body is built with lines, which form layers. In general, the lines from printing appear to be consistent in diameter as shown in Table 2, with values of ~100 µm for each different print composition. The lines appear to be less homogeneous for their diameter for the pure PEEK and the 5 wt% PEEK/HA sample when compared to the 10–30 wt% PEEK/HA samples, shown in Figure A2 (Appendix A).

A small amount of additional HA material incorporated into the PEEK/HA composites is shown to influence the stiffness of the 3D printed samples significantly, with the tensile and flexural modulus shown to increase as the content of HA in the PEEK/HA composites increases, as shown in Figure 3b and c, respectively. The results from the tensile and flexural modulus tests are also reported in Table 2. However, it can be noted that the flexural modulus for the 5 wt% HA loaded PEEK/HA sample is probably slightly higher than expected (in line with the results for the other samples), which may be a consequence of an inhomogeneity in that set of samples. It is also observed here that the tensile yield strain of all the composite materials tested here, as highlighted in Table 2, is between 1.6–2.8%, which is close to human femoral cortical bone performance (~0.67%) [36]. The ultimate tensile strength reported here for the full range of 0–30 wt% HA loading in the 3D printed PEEK/HA composites is found to be between 79.5–94.2 MPa, which are again in close correlation to the performance of human femoral cortical bone (typically ranging from 71–97 MPa) [37]. This is important because the PEEK/HA composites with a higher concentration of HA could be hypothesised to be the most promising material for future implantation as they should encourage a higher degree of osseointegration when implanted and their mechanical properties reported here show a good correlation to human femoral cortical bone. 

### 3.3. Crystallinity

**Figure 4:**
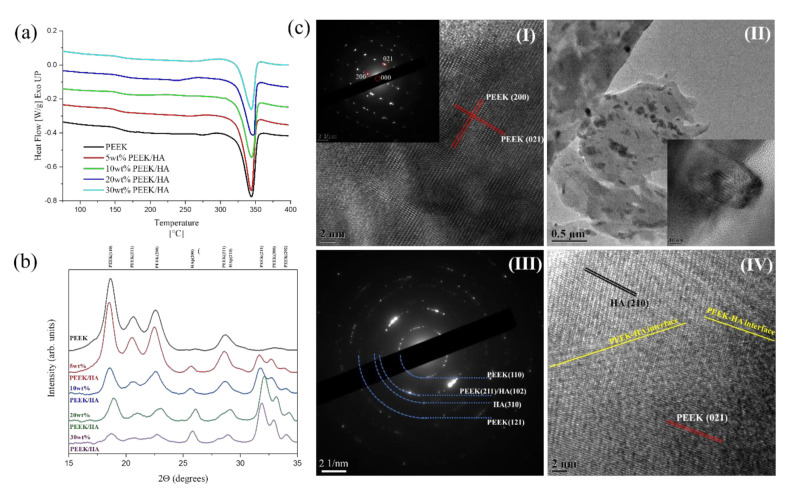
(**a**) First heating scan of DSC 3DP specimen, (**b**) XRD pattern of 3DP samples with various HA content and (**cI**) HRTEM image and corresponding SAED pattern from PEEK, (**cII**) TEM image of 30 wt% PEEK/HA composite, inset showing a HA particle on the surface of PEEK, (**cIII**) The SAED pattern corresponding to Figure (**cII**) and (**cIV**) HRTEM of 30% PEEK/HA showing the lattice fringes corresponding to PEEK and HA.

The results of the Differential Scanning Calorimetry (DSC) first heating scan for the 3D printed samples are characterized by a decreasing crystallinity fraction (X_c_^*DSC^) as the HA content of the PEEK/HA composites increases, as shown in Table 2 and Figure 4. The reason for the significant crystallinity fraction measured here for these 3D printed PEEK/HA composites is thought to be a consequence of the exposure of each single line to the heat derived from the lamps heating the chamber (to around 230 °C), as opposed to the preheated printing bed. It is suggested that the heat from the preheated printing bed cannot be effectively transferred through the polymer as it prints, as the polymer has a low thermal conductivity of 0.26 W/m⸱K [38]. It is noted here in the DSC results that as the HA content increases this does not significantly influence either the glass transition temperature or the melting point, as shown in Figure 4. The calculated grain size is not affected by the filler content and was around 15.6 nm between 0 and 10% wt PEEK/HA. However, when composition reaches a value around 20 wt% PEEK/HA a small decrease in size is observed to 14.6 nm and this trend is kept for 30 wt% PEEK/HA composition with a further decrease to 13.1 nm. The increased level of HA in the 30 wt% PEEK/HA composites could reduce crystal growth of the PEEK in this sample because a large portion of the polymer chains are at the interface with the HA particles and agglomerates. This region is characterised by a reduced ability for relaxation and chain movement within the PEEK, thus limiting its ability to organise into crystalline grains. However, these results help to confirm why there is an increase in tensile and flexural modulus as the wt% of HA increases in the PEEK/HA composites here. The crystallinity fraction calculated from DSC and XRD data, shown in Table 2, shows a significant difference between the two techniques (typically between 9–16% depending on the HA content for 3D printed composite samples). This is because the crystallinity calculated from the XRD analyses (X_c_^*XRD^) considers the total crystallinity of the measured specimen, including both secondary and primary crystallites. The DSC calculation is based on the melting enthalpy for PEEK around 343 °C, and only refers to the melting of the primary crystallites, hence the lower values observed in the DSC results (X_c_^*DSC^) when compared to the XRD results (X_c_^*XRD^) here. Weaker secondary crystals are shown to melt in the region between the glass transition temperature and the melting point, and they are not included in the calculation in the DSC measurements due to difficulties in integrating the results in the region between 160–280 °C. Increasing the HA content of the samples reduces the percentage of the of primary crystallites, and the crystallinity (X_c_^*DSC^) drops from 36.63% to 30.55% for 3D printed pure PEEK and the 30% HA loaded PEEK/HA composites. However, the decrease in crystallinity from the DSC results (X_c_^*DSC^) for the 0–20 wt% HA samples is small (only 2%), with the largest drop observed for the 30 wt% sample recorded as 30.55%, (6.08% less that the pure PEEK sample). In comparison, the total crystallinity calculated from the XRD results (X_c_^*XRD^) increased with increased amounts of HA in the composite material (as shown in Table 2). The HA particles are thought to act as nucleation agents for the crystallisation process, however, this trend is not observed for the 30 wt% HA loaded PEEK/HA sample. It seems that the HA acts as a nucleation agent helping in the initiation of the crystallisation process for the secondary crystals at the beginning of the process (and only up to a certain point of HA loading). However, in the 30 wt% HA loaded PEEK/HA sample, the increased HA content reduces the number of primary crystals by immobilisation of the chain at the interface with the HA particles, not allowing for formation of the expected thicker lamellae structure. This corroborates the findings of the DSC crystallinity calculations (X_c_^*DSC^) and the grain structure calculations.

The X-Ray Diffraction (XRD) pattern for each of the PEEK/HA composites is shown in Figure 4b. For the pure PEEK sample, strong peaks corresponding to various planes have been identified as would be expected for this material. Specifically, peaks at 18.75°, 20.8°, 22.73°, and 28.75° 2θ are attributed to orthorhombic PEEK crystal planes (110), (111), (200) and (211), respectively [39,40]. In the composites containing HA (loaded between 5–30% by weight), additional peaks were observed at 25.88°, 28.96°, 31.80°, 32.94° and 34.08° 2θ correspond to (002), (210), (211), (300) and (202) planes of crystalline HA in accordance with International Centre for Diffraction Data (ICDD) File# 09-0432. Upon increasing the HA concentration in the PEEK/HA composites the intensities of the HA peaks increased as would be expected. The high-resolution Transmission Electron Microscope (HRTEM) image for PEEK is shown in Figure 4(cI) and the corresponding Selected Area Electron Diffraction (SAED) pattern is shown inset. The lattice spacings of 0.38 nm and 0.29 nm corresponding to (200) and (021) planes of PEEK have been clearly identified. From the SAED analysis, same peaks were clearly identified and labelled in the figure. The TEM images and SAED pattern from the 30 wt% HA loaded PEEK/HA composite is shown in Figure 4(cII–IV). The HA particles are well distributed in the PEEK material as highlighted in Figure 4(cII), with the inset diagram here showing a magnified image of HA surrounded by the PEEK matrix. The corresponding SAED pattern for the 30 wt% HA loaded PEEK/HA composite is given in Figure 4(cIII). Diffraction rings, due to multiple crystalline domains, relating to both HA and PEEK were identified and labelled in the image. Due to large fraction of the PEEK crystals in the 30 wt% HA loaded PEEK/HA sample the diffraction patterns corresponding to HA are less intense than those for PEEK. The representative HRTEM from the 30 wt% HA loaded PEEK/HA sample is presented in Figure 4(cIV). As shown, lattice spacings corresponding to HA (210) and PEEK (021) were clearly observed and labelled. The yellow line in Figure 4(cIV) indicates the PEEK/HA interface. Close contact of two types of lattice corresponding to both HA and PEEK suggest that the HA particles are indeed acting as nucleation agents during crystallisation of the semi-crystalline PEEK polymer in this case. 

### 3.4. Morphology

**Figure 5:**
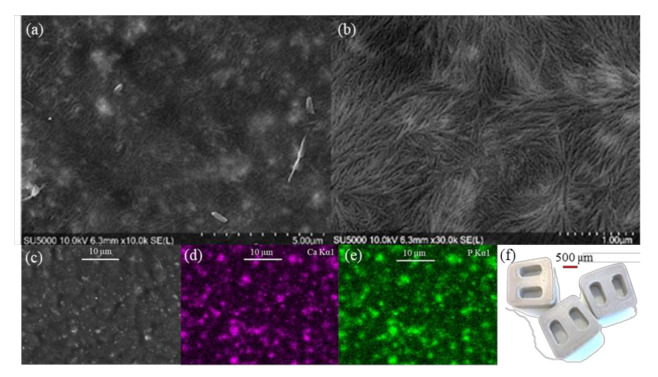
SEM of top 3DP surface for 30 wt% PEEK/HA composite at (**a**) ×10 k and (**b**) ×30 k magnifications. SEM images (**c**) and corresponding (**d**) Ca and (**e**) P elemental maps for 20 wt% PEEK/HA sample, (**f**) photograph of 3DP spinal fusion implants with 20 wt% PEEK/HA.

HA is very well distributed in the 3D printed samples as shown in Figure 5a and Figure A2 (Appendix A) for the Scanning Electron Microscopy (SEM) images. It is possible to notice spherulites on the top layer of the PEEK which are mutually interpenetrating, as can be observed in Figure 5b. The diameter of the observed branches from the high-resolution SEM images is seen to decrease with distance from the centre of the spherulite, with the thickest branch around 155 nm and the thinnest branch measured to be around 28 nm. It seems that the thinnest crystals correspond to the primary crystal block with the size ranging typically from 20–30 nm, and the wider branches are composed with primary and secondary crystalline structures whose size was observed to be between 75–145 nm. The results here are slightly oversized with comparison to the normal published results for PEEK (~35%) [41]. However, the SEM images in the literature were taken after permanganic chemical etching, and that can result in partial dissolution of the crystals thus resulting in smaller observed crystals when compared to the results here, whereby the surfaces of the 3D printed PEEK/HA composites were measured in all cases without any surface treatments of modifications. SEM-EDX mapping of the 3D printed PEEK/HA composites shows that HA is very well distributed in the PEEK matrix, with clear areas of overlapping Ca and P observed across the entire sample as shown in Figure 5d–e for the 20 wt% HA loaded PEEK/HA sample. In combination the µCT results and the SEM-EDX measurements highlight here that the HA material is evenly distributed throughout the bulk of the 3D printed samples and crucially, on the surface of the samples, whereby it can help enhance direct bone apposition and highlights the benefit of applying 3D printing to create PEEK/HA composites for orthopaedic applications. 

## 4. Discussion

The results presented here illustrate for the first time (to the knowledge of the authors) that FFF 3D printing can be used to successfully manufacture PEEK/HA composites up to 30 wt% HA and it addresses a significant gap in the knowledge in this respect. To be suitable for 3D printing, the materials chosen must be firstly printable, they must possess the appropriate structural and mechanical properties for their intended application once they are printed, and they must be biocompatible [42]. The fact that PEEK/HA composites have been successfully directly 3D printed using FFF, shows that the materials are printable under the 3D printing parameters employed here. It is worth noting that beyond 30 wt%, the 3D printing became more problematic and further optimisation would be required to move above this composition. The µCT results for the PEEK/HA filaments shown in Figure 1a highlighted that the 30 wt% PEEK/HA sample had significantly larger agglomerates than the other samples. This has been suggested to be a consequence of the high viscosity of the PEEK matrix at high temperatures during the manufacturing process as reported by Wang et al. [43].

Clearly, the results presented here also show that all the 3D printed PEEK/HA composites (5–30 wt% HA) have mechanical properties in line with human femoral cortical bone (as in the Ultimate Tensile Strength and the Tensile Yield Strain), and that the incorporation of increased levels of HA into the composite bodies provides for increased tensile and flexural moduli. With respect to the tensile modulus of the samples, by comparison the best published results were obtained at chamber temperature 200 °C with a tensile modulus of 4.1 GPa [21,44]. The results shown here are consistent with those results reported in the literature, with the neat matrix (0 wt% HA in PEEK/HA) printed at 230 °C during this study reached a tensile modulus of 4.2 GPa, as highlighted in Table 2. This slight increase was expected due to optimal conditions promoting crystallization of semicrystalline PEEK, with a half-time crystallization minimum at 230 °C. These highlight how enhancements to the mechanical properties are achieved in the PEEK/HA composite when compared to the pure PEEK. On balance, it could be suggested that moving beyond 20–30 wt% HA in the composite results in slightly poorer mechanical properties, however, the 30 wt% HA samples have properties that are still commensurate with human femoral cortical bone and could still have a role to play. Previous works by others have shown that up to 50 wt% HA loaded PEEK/HA composites can be manufactured, although 15–30 wt% seemed to be appropriate compositions to provide enhanced mechanical performance [12]. In other work, calcium sulphate (CaS) was added to PEEK up to 80 wt% CaS, with again the best mechanical performance at 20 wt% CaS [45]. The results in this study highlight that 10 wt% loading with the filler (in our case HA) results in the best mechanical performance of the test specimens with respect to the ultimate tensile strength and the tensile yield strain. It should be noted though that a range of other processing conditions, other than the fraction of filler during FFF can have a dramatic effect on the structure and properties (particularly the mechanical properties) of 3D printed specimens [44]. This includes the temperature of the printing nozzle, the printing bed temperature, and the ambient temperature of the printing chamber. In this work, the nozzle temperature utilised (400 °C) was towards the upper end of what commercial PEEK filament manufacturers would recommend (335–420 °C) [22]. However, the high nozzle temperature applied in this work was necessary to prevent the nozzle blocking, as this is known to occur at lower nozzle temperatures. In addition, the higher temperature of the nozzle provides sufficient melting of the PEEK in the nozzle and the fluidity of the printing material was enhanced delivering enhanced quality prints with lower print void [46].

The ambient temperature of the print chamber was set at 230 °C in this study, which in combination with the nozzle temperature ensured a higher degree of crystallinity for the PEEK matrix in the printed samples as the cooling rate of the materials was slower. Previous work by Wu et al. [47]. highlighted that as the ambient temperature increased, the crystallinity of the samples increased, and the subsequent mechanical performance was enhanced. In general, if the nozzle temperature and ambient temperature are significantly different, this results in warping of the 3D printed specimens. In addition, with a higher chamber temperature this allows for a faster and better crystallisation of the samples [48], and in comparison, to the results reported in this study, with crystallinities for the PEEK matrix reported between 44.59 and 49.91%, these are well above the typical 35% observed for PEEK [22]. Furthermore, in this study higher print-bed temperature (280 °C) was used, which was again at the upper end of what has been explored in the FFF of PEEK specimens in the literature [22]. This again prevents warping as it slows down the cooling rate of the polymer when printed. In combination, the temperature of the print nozzle, the print bed and the print chamber all act collectively to ensure the best quality print, without warping and enhanced crystallinity. They also act to ensure minimal porosity (or print void) in the printed specimens, with all values here reported below 0.732% from the µCT analyses, well below that reported for FFF 3D printed PEEK and PEEK/carbon fibre composites [46,49,50]. It should be noted that the highest porosity was observed for the 5 wt% PEEK/HA sample from the μCT data in A-Table-1, with slightly lower levels observed for all the other samples, except the 20 wt% PEEK/HA sample. These results do not correlate with the observations in the BSE SEM cross sectional images for the same samples in Figure A2. However, the μCT data are taken from a larger sample volume as opposed to a small number of SEM images taken at random from cross-sections of the samples. The μCT data would therefore be a better estimate of the overall porosity of these samples than the supplementary SEM images. The relationship between the porosity of the samples and the wt% HA in the PEEK/HA samples does not follow a definite trend here and is likely a consequence of sampling. It would be proposed that future studies incorporate larger numbers of samples when calculating the porosity using μCT. Despite this, the results here still show favourably low porosity levels in all samples manufactured. The porosity observed in all 3D printed specimens is a consequence of pores formed between lines/layers due to the imperfect deposition process that arises from FFF, as shown in the SEM images of cross sections of the samples Figure A2. This phenomenon has been observed previously when pure PEEK has been 3D printed by FFF [27].

Other parameters that can influence the structure and properties of the FFF 3D printed specimens include the nozzle diameter and layer thickness [44]. In this study a nozzle diameter of 1.0 mm was used, along with a layer thickness of 0.1 mm. This differs significantly than any values reported in the literature, although, the values in the literature are reported for pure PEEK and not PEEK/HA composites. Generally, the best quality prints for PEEK were achieved in the literature with smaller diameter print nozzles (typically in the order 0.4 mm), with the best prints (and best mechanical properties) achieved with a 0.3 mm diameter layer thickness [22,40]. However, when printing composites such as PEEK/HA in this study, a wider diameter nozzle helped to enhance the flow of the composite material from the nozzle, and the lower thickness layer helped to reduce the void space in the prints, given the elevated ambient temperature in the print chamber. The printing layer thickness is also shown to influence the mechanical properties of FFF 3D printed samples, with thicker print layers exhibiting poorer mechanical performance [40]. Further to this the printing orientation, raster angle and printing speed can influence the quality of the prints and the mechanical performance of printed specimens. Generally, horizontally printed samples exhibit the best mechanical performance, along with the lowest print void, and when the raster angle is either 0° or 90° in a horizontal this produces both the best and worst mechanical performance, with a 45° raster angle delivering intermediate mechanical performance between these values [22]. In this study the prints were manufactured at a 45° raster angle, which probably highlights the potential for optimisation of the mechanical properties beyond those achieved here. The printing speed usually has an inverse relationship with mechanical properties (higher speed leads to poorer mechanical properties), which is due to the layers not getting time to diffuse and crystallise properly [40]. In this study a high printing speed was utilised (40 mm per second) and was at higher end of those reported in the literature [22], but as the ambient temperature was generally higher in this work, it is thought that this counterbalances the higher printing speed.

PEEK and HA are both well-established biomaterials that are both currently used for orthopaedic implant devices and through the results demonstrated here, it is not envisaged that FFF of PEEK/HA composites manufactured under similar processing conditions as utilised in this study, will unduly affect their properties, and ultimately their biocompatibility, and prevent their approval for use in orthopaedic implants. However, new 3D printing capabilities also introduce new regulatory challenges, especially for made-to-measure or customisable implant devices, especially with respect to manufacturing quality assurance. Therefore, there is an implicit requirement to fully characterise the properties of the 3D printed materials to provide direct evidence they are safe and effective for clinical use. The extensive µCT, TGA, DSC, FTIR, XRD, SEM-EDX and HRTEM results illustrated here all show that semi-crystalline PEEK/HA composites can be 3D printed and deliver HA particles homogeneously across the uppermost surface of the samples, along with the bulk of the materials, providing both aspects of good mechanical performance and the potential for enhanced bioactivity. Certainly, no unusual results were observed here for any of the PEEK/HA composites, and all the analyses highlighted both chemical and physical properties that were in line with those expected for both PEEK and HA. Therefore, the PEEK/HA composites considered here in this study are all printable and have the appropriate mechanical properties. The presence of HA on the surface could indicate that the materials would show enhanced bioactivity when compared to pure PEEK. However, the true biocompatibility has yet to be tested and is beyond the remit of this initial study of the FFF process and PEEK/HA composites produced from it. A significant advantage of the processing conditions applied during the FFF 3D printing of the PEEK/HA composites here, is the high temperatures of the print nozzle, the print-bed and the print chamber have all led to the PEEK having a higher degree of crystallinity when compared to previous work by others, which has delivered good mechanical performance in all of the samples printed, and consequently this has reduced the need for any post-processing annealing [22]. However, it should be noted that the printing parameters do need to be further optimised here to help to deliver enhanced properties for these PEEK/HA composites.

## 5. Conclusions

In this study 1.75 mm diameter filaments were successfully manufactured from HA and PEEK powders into continuous filaments and 3D printed using a FFF approach on a modified Ultimaker 2+ 3D printer with high temperature capabilities with respect to the heated print bed, the print chamber, and the hot-end printing nozzle. PEEK/HA composites containing HA in the range 0–30 wt% were successfully 3D printed here into solid structures and characterised using mechanical, thermal, physical, and chemical analytical methods. The results here demonstrate the unique spherulite structure of the PEEK visible on the top of the 3D printed specimens, relating to the favourable high temperature crystallization conditions during the 3D printing process, with crystallinities reported between 44.59% and 49.91%, typically higher than the values expected for injection moulded samples. This increased crystallinity significantly enhances the mechanical properties of the 3D printed parts by delivering continuous crystalline domains in all directions. For the 3D printed composites, the mechanical properties such as tensile yield strain of between 1.6%–2.8% and ultimate tensile strength in range 79.5–94.2 MPa are in close relation to the human femoral cortical bone performance. SEM-EDX and µCT analyses show that HA particles are evenly distributed throughout the bulk and across the surface of the native 3D printed samples, which is critical for both enhancing the mechanical properties of the composite materials and providing enhanced osseointegration to ensure more rapid, improved, and stable fixation with bone tissue. The materials could be used in orthopaedic applications, such as spinal fusion devices. The next important step in the development of these materials through FFF 3D printing is to understand their chemical properties, especially on the uppermost surface and to undertake extensive in vitro characterisation to test their biocompatibility. Further studies also need to be conducted to optimise the printing parameters to deliver enhanced properties to these materials.

## Figures and Tables

**Table 1:** 3D Printing parameters.

Description	Value
Nozzle diameter	1.0 mm
Layer Thickness	0.1 mm
Nozzle temperature	400 ℃
Building plate temperature	280 ℃
Chamber temperature	230 ℃
Printing speed	40 mm/s
Raster angle	XY 45°/−45°

**Table 2:** Summary of mechanical and microstructural properties for the 3DP specimens.

	PEEK	5 wt%	10 wt%	20 wt%	30 wt%
Tensile Modulus	4241 ± 189	4379 ± 138	4716 ± 304	5400 ± 302	6110 ± 386
UTS	83.1 ± 6.6	79.5 ± 5.9	94.2 ± 6.1	87.6 ± 12.9	84.9 ± 7.5
Tensile Yield Strain	2.3 ± 0.2	2.1 ± 0.3	2.8 ± 0.1	2.3 ± 0.2	1.6 ± 0.1
Flexural Modulus	4274 ± 506	4827 ± 301	4663 ± 399	5100 ± 505	5686 ± 312
UFS	131 ± 8.9	171 ± 8.4	141 ± 23.0	141 ± 22.3	112 ± 5.69
Δ*H_c_*	47.618	44.515	41.791	37.556	30.547
X_c_^*DSC^ (%)	36.63	35.95	35.37	34.67	30.55
X_c_^*XRD^ (%)	44.59	48.57	49.24	49.91	46.18
Grain size (nm)	15.58	15.55	15.65	14.59	13.10
Line Diameter (µm)	120 ± 27	104 ± 37	100 ± 31	105 ± 27	94 ± 16

Tensile modulus, Ultimate Tensile Strength (UTS), Flexural Modulus and Ultimate Flexural Strength (UFS) in [MPa] unit, Tensile Strain in (%), Heat flow Δ*H_c_* (W/g), X_c_^*DSC^ crystallinity fraction calculated from DSC with excluded filler weight fraction, X_c_^*XRD^ crystallinity fraction calculated from XRD with excluded filler weight fraction, Average grain size (nm). The lines diameter is the measured diameter of the print lines as measured from SEM analyses (µm). The Tensile Modulus, Ultimate Tensile Strength, Tensile Yield Strain, Flexural Modulus, Ultimate Flexural Modulus and Line Diameter are reported as the mean ± standard deviation (where N = 5).

**Figure A3:**
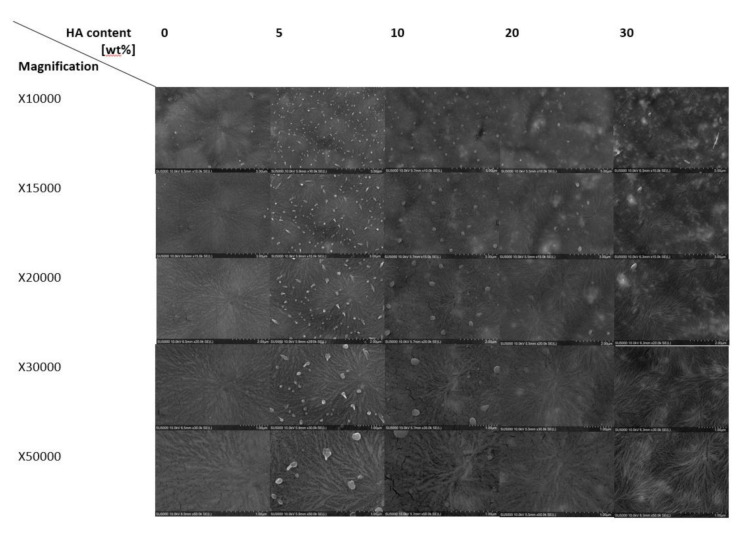
Scanning electron microscope images for rectangular PEEK specimens as 3D printed with HA content between 0–30 wt%.

## Data Availability

The raw/processed data required to reproduce these findings are available from the authors on request.

## Appendix A

**Table A1:** Porosity calculated from CT Scans.

	Porosity—Closed Pores	
Sample	Filaments [%]	3DP Bars [%]
PEEK	0.001	0.272
5 wt% PEEK/HA	0.007	0.732
10 wt% PEEK/HA	0.026	0.478
20 wt% PEEK/HA	0.000	0.039
30 wt% PEEK/HA	0.019	0.497

**Table A2:** Elemental composition of 3D printed materials with the HA content between 0–30 wt% incorporated in PEEK, obtained by SEM-EDX spectrum at 1000 ×magnification.

Atomic Conc (%)	HA Conc wt% (PEEK/HA)				
	0	5	10	20	30
C	83.8	81.6	80.5	76.4	68.1
O	16.2	16.9	16.1	15.5	20.9
Ca	0.0	1.0	2.3	5.5	7.4
P	0.0	0.5	1.1	2.6	3.5

Scherrer equation (Equation (A1)):

(A1) $$\tau =\frac{K\cdot \lambda }{\beta \cdot cos\theta }$$

where: τ—mean size of the crystalline domains, K—shape factor equal 1, λ—X-ray wavelength, β—FWHM, cosθ—Bragg angle

The absolute crystallinity fraction $X_{c}$ can be calculated from the heat of fusion measured by DSC and can be expressed by relation to the heat of fusion of an infinitely thick PEEK crystal (Equation (A2)):

(A2) $$X_{c}=\frac{\Delta H_{m}}{\Delta H_{f}^{0}\cdot w_{polymer}}\cdot 100$$

where: $\Delta H_{f}^{0}$—absolute enthalpy of pure crystal polymer 130 J/g, $\Delta H_{m}$—enthalpy of melting, $w_{polymer}$—weight fraction of polymer matrix.

## References

[1] Berretta S., Evans K.E., Ghita O. (2015). Processability of PEEK, a New Polymer for High Temperature Laser Sintering (HT-LS). Eur. Polym. J..

[2] Milazzo M., Negrini N.C., Scialla S., Marelli B., Farè S., Danti S., Buehler M.J. (2019). Additive Manufacturing Approaches for Hydroxyapatite-Reinforced Composites. Adv. Funct. Mater..

[3] Vaezi M., Black C., Gibbs D.M.R., Oreffo R.O.C., Brady M., Moshrefi-Torbati M., Yang S. (2016). Characterization of New PEEK/HA Composites with 3D HA Network Fabricated by Extrusion Freeforming. Molecules.

[4] Guzzi E.A., Tibbitt M.W. (2019). Additive Manufacturing of Precision Biomaterials. Adv. Mater..

[5] Noiset O., Schneider Y.J., Marchand-Brynaert. J. (1999). Fibronectin adsorption or/and covalent grafting on chemically modified PEEK film surfaces. Biomater. Sci. Polym. E.

[6] Ozeki K., Masuzawa T., Aoki H. (2017). Fabrication of hydroxyapatite thin films on polyetheretherketone substrates using a sputtering technique. Mater. Sci. Eng. C.

[7] Lee J.H., Jang H.L., Lee K.M., Baek H.R., Jin K., Hong K.S. (2013). In vitro and in vivo evaluation of the bioactivity of hydroxyapatite-coated polyetheretherketone biocomposites created by cold spray technology. Acta Biomater..

[8] Yuan B., Cheng Q., Zhao R., Zhu X., Yang X., Yang X., Zhang K., Song Y., Zhang X. (2018). Comparison of osteointegration property between PEKK and PEEK: Effects of surface structure and chemistry. Biomaterials.

[9] Almasi D., Iqbal N., Sadeghi M., Sudin I., Rafiq M., Kadir A., Kamarul T. (2016). Preparation Methods for Improving PEEK’s Bioactivity for Orthopedic and Dental Application: A Review. Int. J. Biomater..

[10] Ma R., Guo D. (2019). Preoperative sleep quality affects postoperative pain and function after total joint arthroplasty: A prospective cohort study. J. Orthop. Surg. Res..

[11] Zhong G., Vaezi M., Mei X., Liu P., Yang S. (2019). Strategy for Controlling the Properties of Bioactive Poly-EtherEther-Ketone/Hydroxyapatite Composites for Bone Tissue Engineering Scaffolds. ACS Omega.

[12] Oladapo B.I., Zahedi S.A., Ismail S.O. (2020). 3D printing of PEEK–cHAp scaffold for medical bone implant. Bio-Des. Manuf..

[13] Zhang Y., Hao L., Savalani M.M., Harris R.A., di Silvio L., Tanner K.E. (2009). In vitro biocompatibility of hydroxyapatite-reinforced polymeric composites manufactured by selective laser sintering. J. Biomed. Mater. Res. A.

[14] Yu S., Hariram P.K., Kumar R., Cheang P., Aik K.K. (2005). In vitro apatite formation and its growth kinetics on hydroxyapatite/polyetheretherketone biocomposites. Biomaterials.

[15] Ma R., Fang L., Luo Z. (2014). Mechanical performance and in vivo bioactivity of functionally graded PEEK–HA biocomposite materials. J. Sol.-Gel Sci. Technol..

[16] Baştan F.E. (2020). Fabrication and characterization of an electrostatically bonded PEEK-hydroxyapatite composites for biomedical applications. J. Biomed. Mater. Res..

[17] Rousseau M.A., Lazennec J.Y., Saillant G. (2007). Circumferential arthrodesis using PEEK cages at the lumbar spine. J. Spinal. Disord. Tech..

[18] Sun F., Shen X., Zhou N., Gao Y., Guo Y., Yang X., Wu G. (2019). A speech bulb prosthesis for a soft palate defect with a polyetherketoneketone (PEKK) framework fabricated by multiple digital techniques: A clinical report. J. Prosthet. Dent..

[19] Dong L., Jun F., Hongbin F., Dichen L., Enchun D., Xin X., Ling W., Zheng G. (2018). Application of 3D-printed PEEK scapula prosthesis in the treatment of scapular benign fibrous histiocytoma: A case report. J. Bone Oncol..

[20] Berretta S., Evans K., Ghita O. (2018). Additive manufacture of PEEK cranial implants: Manufacturing considerations versus accuracy and mechanical performance. Mater. Des..

[21] Yang C., Tian X., Li D., Cao Y., Zhao F., Shi C. (2017). Influence of thermal processing conditions in 3D printing on the crystallinity and mechanical properties of PEEK material. J. Mater. Process. Tech..

[22] Cicala G., Latteri A., del Curto B., Russo A.L., Recca G., Farè S. (2017). Engineering thermoplastics for additive manufacturing: A critical perspective with experimental evidence to support functional applications. J. Appl. Biomater. Funct. Mater..

[23] Han X., Sharma N., Xu Z., Scheideler L., Geis-Gerstorfer J., Rupp F., Thieringer F.M., Spintzyk S.J. (2019). An In Vitro Study of Osteoblast Response on Fused-Filament Fabrication 3D Printed PEEK for Dental and Cranio-Maxillofacial Implants. J. Clin. Med..

[24] Singh S., Prakash C., Ramakrishna S. (2019). 3D printing of polyether-ether-ketone for biomedical application. Eur. Polym. J..

[25] Zanjanijamm R., Major I., Lyons J.G., Lafont U., Devine D.M. (2020). Fused Filament Fabrication of PEEK: A Review of Process-Structure-Property Relationships. Polymers.

[26] Velasco-Hogan A., Xu J., Meyers M.A. (2018). Additive Manufacturing as a Method to Design and Optimize Bioinspired Structures. Adv. Mater..

[27] Golbang A., Harkin-Jones E., Wegrzyn M., Campbell G., Archer E., Mcilhagger A. (2020). Production and characterization of PEEK/IF-WS2 nanocomposites for Additive Manufacturing: Simultaneous improvement in processing characteristics and material properties. Addit. Manuf..

[28] Vasconcelos G.C., Mazur R.L., Ribeiro B., Botelho E.C., Costa M.L. (2014). Evaluation of decomposition kinetics of poly (ether-ether-ketone) by thermogravimetric analysis. Mater. Res..

[29] Rodzeń K., Strachota A., Ribot F., Matejka L., Kovářová J., Trchová M., Slouf M. (2015). Reactivity of the tin homolog of POSS, butylstannoxane dodecamer, in oxygen-induced crosslinking reactions with an organic polymer matrix: Study of long-time behavior. Polym. Degrad. Stab..

[30] Papageorgiou D.G., Roumeli E., Chrissafis K., Lioutas C., Triantafyllidis K., Bikiaris D., Boccaccini A.R. (2014). Thermal degradation kinetics and decomposition mechanism of PBSu nanocomposites with silica-nanotubes and strontium hydroxyapatite nanorods. Phys. Chem. Chem. Phys..

[31] Amaral M., Lopes M.A., Silva R.F., Santos D.J. (2002). Densification route and mechanical properties of Si3N4-bioglass biocomposites. Biomaterials.

[32] Rodzeń K., Strachota A., Raus V., Pavlova E. (2017). Behavior of Tin-Based “Super-POSS” Incorporated in Different Bonding Situations in Hybrid Epoxy Resins. Polym. Degrad. Stab..

[33] Strachota B., Strachota A., Horodecka S., Steinhart M., Kovářová J., Pavlova E., Ribot F. (2019). Polyurethane nanocomposites containing the chemically active inorganic Sn-POSS cages. React. Funct. Polym..

[34] Tan S., Su A., Luo J., Zhou E. (1999). Crystallization kinetics of poly(ether ether ketone) (PEEK) from its metastable melt. Polymer.

[35] Tardif X., Pignon B., Boyard N., Schmelzer J.W.P., Sobotka V., Delaunay D., Schick C. (2014). Experimental study of crystallization of PolyEtherEtherKetone (PEEK) over a large temperature range using a nano-calorimeter. Polym. Test..

[36] Wolframa U., Schwiedrzik J. (2016). Post-yield and failure properties of cortical bone. Bonekey Rep..

[37] Wall J.C., Chatterji S.K., Jeffery J.W. (1979). Age-related changes in the density and tensile strength of human femoral cortical bone. Calcif. Tiss. Intl..

[38] Riviere L., Causse N., Lonjon A., Dantras E., Lacabanne C. (2016). Specific heat capacity and thermal conductivity of PEEK/Ag nanoparticles composites determined by Modulated-Temperature Differential Scanning Calorimetry. Polym. Degrad. Stab..

[39] Wang Y., Chen B., Evans K., Ghita O. (2018). A transfer learning approach for microstructure reconstruction and structure-property predictions. Sci. Rep..

[40] Wang S., Wang J., Liu T., Mo Z., Zhang H., Yang D., Wu Z. (1997). The crystal structure and drawing-induced polymorphism in poly (aryl ether ketone) s, 2. Poly (ether ether ketone ketone), PEEKK. Macromol. Chem. Phys..

[41] Wang Y., Beard J.D., Evans K.E., Ghita O. (2016). Unusual crystalline morphology of poly aryl ether ketones (PAEKs). RSC Adv..

[42] Xie Z., Gao M., Lobo A.O., Webster T.J. (2020). 3D Bioprinting in Tissue Engineering for Medical Applications: The Classic and the Hybrid. Polymers.

[43] Wang L., Weng L., Song S., Sun Q. (2010). Mechanical properties and microstructure of polyetheretherketone–hydroxyapatite nanocomposite materials. Mater. Lett..

[44] Fan D., Staufer U., Accardo A. (2019). Engineered 3D polymer and hydrogel microenvironments for cell culture applications. Bioengineering.

[45] Hughes A.B., Grover L.M. (2017). Characterisation of a novel poly (ether ether ketone)/calcium sulphate composite for bone augmentation. Biomater. Res..

[46] Wang P., Zou B., Xiao H., Ding S., Huang C. (2019). Effects of printing parameters of fused deposition modeling on mechanical properties, surface quality, and microstructure of PEEK. J. Mater. Process. Technol..

[47] Wu W.Z., Geng P., Zhao J., Zhang Y., Rosen D.W., Zhang H.B. (2014). Manufacture and thermal deformation analysis of semicrystalline polymer polyether ether ketone by 3D printing. Mater. Res. Innov..

[48] Geng P., Zhao J., Wu W., Wang Y., Wang B., Wang S., Li G. (2018). Effect of thermal processing and heat treatment condition on 3D printing PPS properties. Polymers.

[49] Basgul C., MacDonald D.W., Siskey R., Kurtz S.M. (2020). Thermal localization improves the interlayer adhesion and structural integrity of 3D printed PEEK lumbar spinal cages. Materialia.

[50] Berretta S., Davies R., Shyng Y.T., Wang Y., Ghita O. (2017). Fused Deposition Modelling of high temperature polymers: Exploring CNT PEEK composites. Polym. Test..

